# Ecophysiology of coral reef primary producers across an upwelling gradient in the tropical central Pacific

**DOI:** 10.1371/journal.pone.0228448

**Published:** 2020-02-04

**Authors:** Maggie D. Johnson, Michael D. Fox, Emily L. A. Kelly, Brian J. Zgliczynski, Stuart A. Sandin, Jennifer E. Smith

**Affiliations:** Center for Marine Biodiversity and Conservation, Scripps Institution of Oceanography, University of California San Diego, San Diego, California, United States of America; Australian Bureau of Agricultural and Resource Economics and Sciences, AUSTRALIA

## Abstract

Upwelling is an important source of inorganic nutrients in marine systems, yet little is known about how gradients in upwelling affect primary producers on coral reefs. The Southern Line Islands span a natural gradient of inorganic nutrient concentrations across the equatorial upwelling region in the central Pacific. We used this gradient to test the hypothesis that benthic autotroph ecophysiology is enhanced on nutrient-enriched reefs. We measured metabolism and photophysiology of common benthic taxa, including the algae *Porolithon*, *Avrainvillea*, and *Halimeda*, and the corals *Pocillopora* and *Montipora*. We found that temperature (27.2–28.7°C) was inversely related to dissolved inorganic nitrogen (0.46–4.63 *μ*M) and surface chlorophyll *a* concentrations (0.108–0.147 mg m^-3^), which increased near the equator. Contrary to our prediction, ecophysiology did not consistently track these patterns in all taxa. Though metabolic rates were generally variable, *Porolithon* and *Avrainvillea* photosynthesis was highest at the most productive and equatorial island (northernmost). *Porolithon* photosynthetic rates also generally increased with proximity to the equator. Photophysiology (maximum quantum yield) increased near the equator and was highest at northern islands in all taxa. Photosynthetic pigments also were variable, but chlorophyll *a* and carotenoids in *Avrainvillea* and *Montipora* were highest at the northern islands. Phycobilin pigments of *Porolithon* responded most consistently across the upwelling gradient, with higher phycoerythrin concentrations closer to the equator. Our findings demonstrate that the effects of in situ nutrient enrichment on benthic autotrophs may be more complex than laboratory experiments indicate. While upwelling is an important feature in some reef ecosystems, ancillary factors may regulate the associated consequences of nutrient enrichment on benthic reef organisms.

## Introduction

The availability of inorganic nutrients in the environment influences organismal physiology and can have cascading effects on ecosystem-scale processes [1]. Both natural and anthropogenic sources of nutrients can change community structure and function by facilitating growth of some species over others, in addition to increasing an ecosystem’s energetic foundation, capacity to support greater biomass [2], and trophic complexity [3]. In marine systems, the majority of research on nutrient enrichment has focused on the effects of anthropogenic inputs, including the process of eutrophication, which can overwhelm and fundamentally alter coastal habitats [4]. However, natural inputs are also important in nearshore systems, and can be linked to large-scale oceanographic processes such as upwelling, eddies, and internal tides [5]. Though the effects of coastal upwelling have been relatively well-studied in temperate ecosystems [6], less is known about how inorganic nutrient input from upwelling affects classically oligotrophic systems such as tropical coral reefs.

Persistent upwelling caused by long-standing currents [7], or periodic upwelling caused by episodic processes (e.g., internal tides) [8], deliver subthermocline water enriched in dissolved inorganic nitrate, phosphate, and carbon to the ocean surface [5]. These deep, cooler waters mix with warmer surface waters and replenish depleted inorganic nutrients that are important for regulating biological activity [9,10]. Such interactions of environmental variables with organismal function are referred to as ecophysiology. Large-scale oceanographic processes, like upwelling, can influence the ecophysiology of resident organisms, particularly those that require inorganic nutrients for essential biological processes. For example, nutrient availability directly influences autotroph photosynthesis and respiration. Alterations to the nutrient landscape resulting from natural or anthropogenic enrichment can have cascading effects on broader ecosystem processes by altering primary production [11,12], by favoring growth of some species over others [13], and, ultimately, by affecting how much energy is available to higher trophic levels [3]. Thus, understanding how the delivery of essential nutrients by upwelling can influence autotroph ecophysiology will shed light on how natural oceanographic processes shape ecosystem structure and function.

Autotrophs are particularly sensitive to availability of inorganic nutrients, because dissolved inorganic nitrogen (DIN) and phosphorous (DIP) are requisite in the synthesis and use of photosynthetic machinery [11,14]. Though marine photosynthesis is generally not limited by the availability of carbon, due to the abundant supply of inorganic carbon in seawater and carbon-concentrating mechanisms in many autotrophs [15], it is often limited by the availability of inorganic nutrients [11]. For example, nitrate or phosphate limitation decreases productivity and growth in numerous autotrophic taxa [1,16] including phytoplankton [17], microalgae [18], and macroalgae [19]. Accordingly, greater availability of DIN and DIP enhances photosynthesis and generally increases marine primary production [20], and the input of inorganic nutrients from upwelling consistently increases productivity of phytoplankton communities in the surface ocean [10,21]. Though a positive relationship between autotroph physiology and nutrient availability is well established, there are instances where ancillary abiotic factors, such as iron limitation, can constrain photosynthesis and primary production of algae in nutrient replete conditions [22,23].

Given the fundamental role of nutrients in marine photosynthesis and autotroph physiology, ecosystems depleted in nutrients and dominated by autotrophs have the potential to respond strongly to nutrient inputs. Tropical coral reefs are typically oligotrophic, with the benthos dominated by highly productive macroalgae and corals that provide the primary source of carbon fixation for the ecosystem [24]. Corals are considered mixotrophs, because their energy comes from consuming organic particulates (i.e., heterotrophy) and from utilizing carbon fixed by photosynthesis of endosymbiotic microalgae in the family Symbiodiniaceae (i.e., autotrophy). Yet, at an ecosystem scale, they typically function as autotrophs, because the majority (up to 100%) of the daily energetic requirements of most corals is derived from photosynthesis [25, 26]. Hereafter, we consider corals from an autotrophic perspective, grouping them (and their endosymbiotic microalgae) with algal taxa common on coral reefs. In tropical ecosystems with an abundance of autotrophs, such as coral reefs, the availability of DIN and DIP is perhaps the most important factor constraining photosynthesis and primary production, because light is abundant and temperatures are relatively stable [27]. Coral reefs are, thus, an ideal study system to explore the effects of natural nutrient inputs from upwelling in a typically low nutrient ecosystem.

Upwelling has been documented on coral reefs across ocean basins, ranging from the Florida Keys [8] and the Colombian Caribbean [28] to the Seychelles [29] and the Great Barrier Reef [30]. The tropical central Pacific, where coral reefs exist around remote islands and atolls, is another region where upwelling likely plays an important role [31,32]. Equatorial upwelling is a persistent oceanographic process, caused by divergence of Ekman transport to the equator [7], that creates a region from ~8°N to 8°S of the equator [33] where surface-ocean inorganic nutrient concentrations are higher than oligotrophic tropical gyres [34]. The nutrient input from upwelling supports high levels of primary production in equatorial Pacific surface waters, as evidenced by increased surface-ocean productivity closer to the equator [10,21,35]. While patterns of phytoplankton productivity in the equatorial Pacific are well established, less is known about how the associated increase in inorganic nutrients effects the ecophysiology of benthic autotrophs.

**Fig 1:**
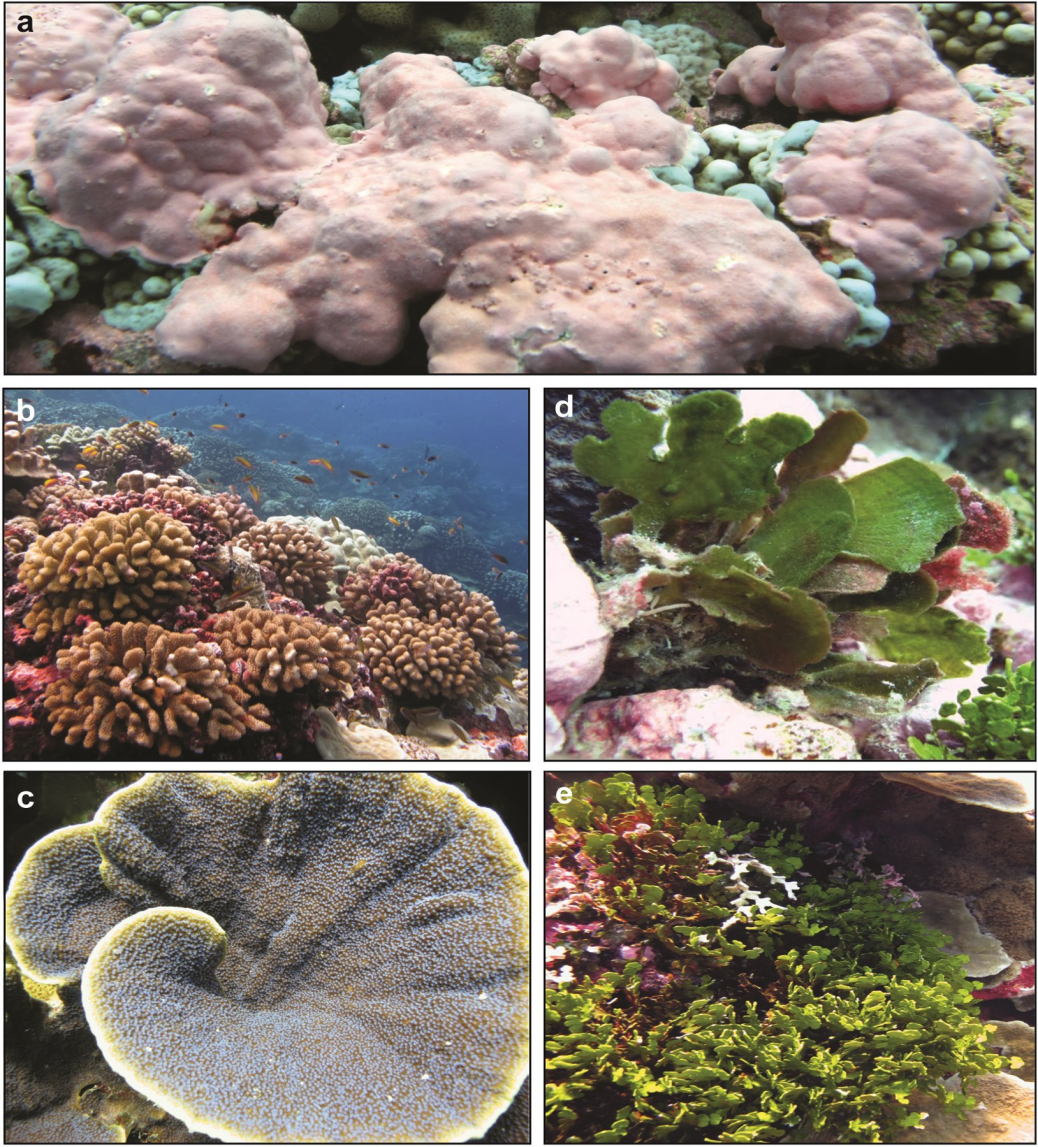
Coral and algae of the Southern Line Islands. Common genera of corals and algae across the Southern Line Islands in the Republic of Kiribati, central Pacific. (a) The crustose coralline alga, *Porolithon* sp. and (b) the corals *Pocillopora meandrina* and (c) *Montipora aequituberculata* were collected from all five islands. (d) The fleshy green alga *Avrainvillea amadelpha* was found only at Vostok and Malden, and (e) the calcareous green alga, *Halimeda* sp., was found at Flint, Millennium and Starbuck.

We used a natural gradient of surface ocean productivity and nutrient availability across the Southern Line Islands (SLI) to explore how upwelling-driven nutrient enrichment influenced the ecophysiology of benthic autotrophs common on coral reefs (Fig 1). The goals of this study were to, 1) characterize key environmental characteristics associated with upwelling across the SLI archipelago and, 2) to quantify corresponding changes in the ecophysiology of common benthic autotrophs. We hypothesized that metabolism (i.e., photosynthesis and respiration) and photophysiology (i.e., maximum quantum yield and photosynthetic pigment content) of abundant coral and algal genera would increase across the SLI with greater exposure to upwelling. We further predicted that metabolic rates and pigment concentrations would increase (indicating enhancement), with increasing proximity to the equator. By understanding patterns in ecophysiology of key benthic autotrophs across the SLI, we can consider how patterns in surface-ocean phytoplankton productivity relate to patterns in benthic productivity on coral reefs.

## Materials and methods

### Site description

This study was conducted on a 2013 expedition to the SLI aboard the *M/Y Hanse Explorer* under a Scientific Research Permit granted to SAS from the Republic of Kiribati, Environment and Conservation Division (permit #021113). The five uninhabited islands of the SLI span ~900 km from Malden Island in the north to Flint Island in the south (S1 Table). At 4.0° and 5.6° south of the equator, Malden and Starbuck Islands, respectively, are situated within the equatorial upwelling region that spans ~8°N to 8°S of the equator [33]. The remaining atoll and two islands (Millennium Atoll, Vostok Island, Flint Island) lie at increasing distances from the upwelling region (S1 Table), with Flint Island at the southernmost end of the archipelago. The coral reefs of the SLI are characterized by high biomass of herbivorous fish and top predators [36] and are dominated by reef-building coralline algae and corals [37].

### Remote sensing

To estimate surface-ocean primary production across the SLI, we used the eight-day 0.0417° (4 km) spatial resolution product of chlorophyll *a* (chl *a*, mg mg^-3^) derived from the Moderate Resolution Imaging Spectroradiometer (https://modis.gsfc.nasa.gov/about/) (sensu Gove et al. (2013)[38]). Monthly mean chl *a* data were generated for each island and averaged over the period of September–December 2013 to provide an integrated estimate of surface-ocean primary production around each island over the period of the cruise. The oceanographic conditions during this time are most relevant to the ecophysiology of the organisms studied [39] and provide a reliable estimate of annual conditions across the SLI [40]. While these estimates of surface-ocean production are measured in offshore waters adjacent to the SLI reefs, they can be used to reliably infer reef-scale patterns such as benthic community structure [41] and ecosystem calcification [42]. To confirm that large-scale patterns of surface primary production track in situ nutrient environments across the SLI, we also collected discrete water samples from above the reef benthos as described below.

### Environmental parameters

Water samples were collected for nutrient analyses at each island over three separate sampling intervals. During a cruise to the SLI in 2009, water samples were collected in triplicate from ~ 1 m above the benthos at 1–3 sites per island (referred to as 2009 in situ) [31]. During the 2013 cruise, three water samples were collected from ~1 m above the benthos every 5 m from 5–25 m depth (referred to as 2013 in situ) [40]. There were no differences in nutrient concentrations across depths in 2013, thus all depths were pooled for a more robust estimate of environmental conditions across the reef slope of an island on a given day (*n* = 9–12, per island). Additionally, triplicate water samples were collected from the bulk seawater used during metabolic incubations at each island (referred to as 2013 incubation). Seawater samples were filtered through 1.2 *μ*M GF/C filters (Whatman) and frozen at -20°C. Samples from 2009 and 2013 in situ were analyzed at the University of Hawaii Hilo EPSCoR analytical laboratory and 2013 incubation samples were analyzed at the University of California Santa Barbara Marine Science Institute Analytical lab. Seawater was analyzed for total DIN, which includes nitrate (NO_3_^-^), nitrite (NO_2_^-^) and ammonium (NH_4_^+^), and for DIP. Replicate samples were averaged by island for each sampling period.

To assess in situ temperature we deployed 6–8 autonomous sensors (Manta 2, Eureka Environmental Engineering) at 10 m depth for 3–4 days per island (5 min sampling interval). Daily mean temperatures were calculated across sensors to determine daily and overall site means. Photosynthetically active radiation (PAR) was measured adjacent to the instrument with a 4π quantum sensor (LI-193) attached to a LiCor LI-1400 meter in an underwater housing. We measured PAR every minute for 2–3 days and calculated an average light intensity during the period of maximum irradiance and peak photosynthesis per day (10:00–14:00) [43], and then a daily average per island. All deployments occurred on sunny days, except for one cloudy day at Vostok.

### Specimen collection

Two species of coral and up to three genera of algae were collected at 10 m depth from unshaded microhabitats at each island (adjacent to sensors), with four individuals of each taxa (Fig 1 and S1 Table). After collection, samples were held in buckets with ambient seawater under shaded, ambient light until physiological incubations. Water was regularly replenished with new ambient seawater. In general, we sampled the most abundant coral and algal taxa on a given island, but due to biogeographic differences we were unable to collect the same species at all islands (S1 Table). At all islands, we collected similar sized fragments of the branching coral *Pocillopora meandrina*, the plating coral *Montipora aequituberculata*, and the crustose coralline alga (CCA) *Porolithon* sp. (5–8 cm diameter/height). Calcareous algae in the genus *Halimeda* were present only at Flint, Starbuck, and Millennium. We collected 8 cm long fragments of the most abundant species at each of the three islands (Flint: *H*. *opuntia*, Starbuck: *H*. *micronesica*, Millennium: *H*. *taenicola*). We consider *Halimeda* at the genus level because photophysiology and response to higher nutrient concentrations are similar across species within the genus [44,45]. Abundance of fleshy macroalgae is exceedingly low across the SLI [37], and the fleshy green alga *Avrainvillea amadelpha* was present only at Malden and Vostok.

### Metabolism

Physiological studies were conducted aboard the *M/Y Hanse Explorer* over 3–4 days at each island, with incubations commencing within 1–2 hrs after sample collection. Photosynthesis and respiration rates were determined by measuring oxygen exchange under saturating irradiance and total darkness, respectively, using sealed metabolic incubation chambers and optical dissolved oxygen (DO) probes (Hach Intellical LDO101) following the exact methods of Johnson et al. (2017) [46]. All incubations of a given species (*n* = 4, per species), and a blank control, were run simultaneously in a temperature controlled bath. Each chamber contained one specimen (or seawater control) and a magnetic stir bar. No more than 2 taxa were incubated on a given day. Samples were kept in ambient conditions for 1–3 hrs before the start of incubations, and were acclimated to incubation chambers for 5–10 min before the start of measurements. Saturating irradiance (~700 *μ*M quanta m^2^ s^-1^) was supplied by two LED light fixtures (Aqua Illumination, Hydra) suspended directly above the incubation chambers. Light levels inside incubation chambers were measured with a submerged 4π quantum sensor (LI-193) and a LiCor LI-1400 meter. Due to a probe malfunction, no oxygen production data were collected for *Porolithon* at Starbuck.

Photosynthesis and respiration were calculated as the linear slope of oxygen concentration over the duration of the incubation (45 min), and the blank for each set of incubations was subtracted from the rates of respective replicates to account for background changes in oxygen. Oxygen production in the light was assumed to represent net photosynthesis and oxygen consumption in the dark to represent respiration. To estimate total oxygen produced during incubations, or gross photosynthesis, respiration rates were added to net photosynthesis. For consistency, rates across all taxa were normalized to surface area and are expressed as *μ*mol O_2_ cm^-2^ hr^-1^. Surface area was determined for coralline fragments by foil-wrapping following March (1970) [47], for corals by wax dipping following Stimson and Kinzie (1991) [48], and for algae by planar image analysis using ImageJ.

### Maximum quantum yield

Maximum quantum yield is a useful proxy for photosynthetic efficiency because it is proportional to the rate of electron transport, and it provides a convenient, non-invasive, and instantaneous estimate of the performance of the photosynthetic machinery in both algae and corals [49]. Maximum quantum yield was measured with a red Pulse Amplitude Modulation Fluorometer (Diving PAM, Walz). Samples were dark adapted for 2 hours after respiration measurements. To account for variation in fluorescence-based maximum quantum yield (F_v_/F_m_) measurements within individuals, we calculated an average of three haphazardly selected points on each individual from approximately the same region (~ 2 cm from the tip/edge). Measuring light intensity was minimized to avoid actinic effects (F_0_ = 300–500) and gain was minimized to avoid amplifying noise [50]. Saturation intensity (8) and saturation pulse width (0.8) were constant for all measurements. Due to logistical difficulties, no measurements were taken at Flint.

### Photosynthetic pigments

Samples were frozen at -20°C after physiological assessments and transported to Scripps Institution of Oceanography for pigment analyses. Extractions of chl *a* and carotenoids followed the methods and equations of Moran and Porath (1980) [51] and Wellburn (1994) [52]. In brief, chl *a* and carotenoids were extracted from three intact subsamples of each *Halimeda* and *Avrainvillea* individual in *N*,*N*-Dimethylformamide (DMF). Pigments were extracted in total darkness at 4°C for 24 hrs. The extract was then centrifuged and the supernatant was analyzed spectrophotometrically with a diode array spectrophotometer (Agilent, UV-vis 8453) with 1 nm resolution. For the coral samples, coral tissue was first removed from the skeleton with an airbush, and the blastate was homogenized. The endosymbiotic algal fraction was isolated from the host animal tissue, and chl *a* and carotenoids were extracted from the algal fraction in DMF as described above. Two subsamples from each coral individual were analyzed.

Pigments were extracted from the coralline alga, *Porolithon*, with procedures adapted from Payri et al. (2001) [53] and Kursar and Alberte (1983) [54]. Water soluble phycobilin pigments were extracted first using 0.01 M phosphate buffer. Two 1-cm^2^ punches were collected from each coralline algal sample and then each subsample was ground separately in the dark and over ice using a mortar and pestle. Pigments were extracted in darkness at 4°C for 24 hrs. Samples were then centrifuged and the supernatant was analyzed for allophycocyanin, phycocyanin, and phycoerythrin content based on the equations of Kursar and Alberte (1983) [54]. The remaining pellets were then processed for chl *a* and carotenoids in DMF following the procedure above. Subsamples from algal pigment extractions were averaged for each individual. *Porolithon* and coral pigments were normalized to subsample surface area and macroalgal pigments were normalized to subsample wet weight.

### Statistical analyses

All variables met assumptions of normality and homogeneity of variances, determined by visual inspection of residuals using q-q plots and Shapiro-Wilks tests and Levene’s test, respectively. Mean environmental parameters were analyzed across islands with a one-way ANOVA with island as a fixed factor. In situ temperatures and remotely sensed chl *a* were compared using daily and 8-day mean values, respectively. Inorganic nutrient concentrations were analyzed separately for each sampling interval (2009 in situ, 2013 in situ, 2013 incubation). Daily mean light intensity was compared using values between 10:00–14:00. Each response variable was analyzed separately by genus with a one-way ANOVA with island as a fixed factor. Where significant differences were detected, Tukey’s HSD identified differences among islands. All statistical analyses were conducted in R [55].

## Results

### Environmental parameters

**Fig 2:**
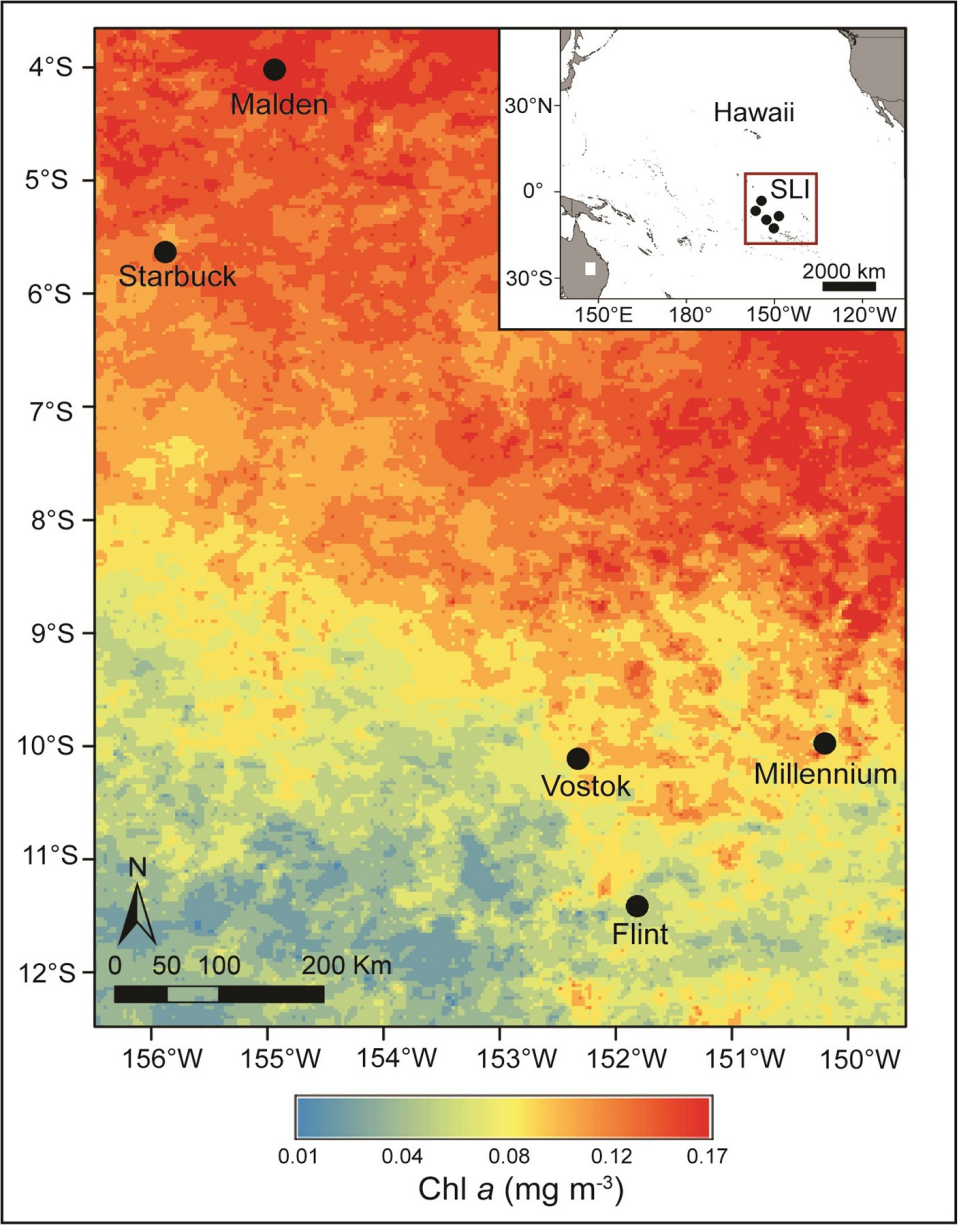
Surface-ocean chl *a* during cruise. Average surface-ocean chl *a* concentrations increased from south to north across the Southern Line Islands over four months encompassing the research expedition. Surface chl *a* (mg mg^-3^) was estimated from the eight-day 0.0417° (4 km) spatial resolution product derived from the Moderate Resolution Imaging Spectroradiometer, and concentrations ranged from 0.01 (blue) to 0.17 mg mg^-3^ (red) across the archipelago.

Surface chl *a* concentrations were higher near the equator, and lower farther from the equator (Fig 2 and S2 Table). From Sept—Dec 2013, mean surface chl *a* was highest at Malden, lowest at Flint, and similar across the three middle islands. Malden surface water chl *a* concentrations were 10% higher than Starbuck, while Starbuck, Millennium and Vostok were 15–20% higher than Flint (Table 1).

**Table 1:** Environmental parameters across the Southern Line Islands, with islands listed from south to north.

Island	Year	Temp (°C)	PAR^§^	Chl *a* (mg m^-3^)	DIN (*μ*M)	DIP (*μ*M)
Flint	2013 in situ	28.50 ± 0.04	608 ± 3	0.108 ± 0.003	0.46 ± 0.01	0.13 ± 0.003
	2013 incubation 2009 in situ				1.16 ± 0.16 0.78 ± 0.02	0.20 ± 0.005 0.15 ± 0.003
Vostok	2013 in situ	28.70 ± 0.01	428 ± 43	0.128 ± 0.001	0.93 ± 0.05	0.16 ± 0.01
	2013 incubation 2009 in situ				1.62 ± 0.33 1.51 ± 0.35	0.23 ± 0.02 0.16 ± 0.003
Millennium	2013 in situ	28.70 ± 0.02	647 ± 4	0.125 ± 0.004	0.95 ± 0.08	0.16 ± 0.01
	2013 incubation 2009 in situ				1.18 ± 0.13 2.03 ± 0.06	0.22 ± 0.02 0.21 ± 0.01
Starbuck	2013 in situ	28.30 ± 0.01	798 ± 12	0.133 ± 0.001	3.86 ± 0.05	0.42 ± 0.03
	2013 incubation 2009 in situ				4.50 ± 0.04 3.59 ± 0.38	0.35 ± 0.02 0.23 ± 0.01
Malden	2013 in situ	27.20 ± 0.05	884 ± 1	0.147 ± 0.003	4.63 ± 0.13	0.44 ± 0.01
	2013 incubation 2009 in situ				5.15 ± 0.06 3.87 ± 0.32	0.51 ± 0.07 0.27 ± 0.02

Island-scale mean (± SE) environmental parameters across the Southern Line Islands. In situ temperature and photosynthetically active radiation (PAR) were measured with autonomous sensors for 2–4 days per island. Average PAR at peak irradiance (between 1000–1400) is presented, as these values most closely resemble intensities used in lab incubations. Chl *a* concentrations were derived from satellite data spanning a four-month window around the expedition (Sept—Dec 2013). In situ water samples were collected for analysis of dissolved inorganic nitrogen (DIN) and dissolved inorganic phosphorous (DIP) from one site per island in 2013 (*n* = 9–12), and in triplicate from 3–6 sites per island during a research cruise in 2009.

^§^*μ*mol quanta^-1^ m^-2^ s^-1^.

Mean daily in situ temperatures decreased significantly across the SLI based on latitude (S2 Table), with coolest temperatures closest to the equator. Temperatures were similarly warm at Vostok and Millennium, at 28.70 ± 0.01°C and 28.70 ± 0.02°C, respectively, and decreased to 27.20 ± 0.05°C at Malden (Table 1). Average daily PAR levels did not vary significantly across islands (S2 Table), though the lowest daily average intensity between the hours of 10:00–14:00 was at Vostok on a cloudy day (428 ± 43) and the highest was at Malden (884 ± 1) (Table 1).

In situ water samples from 2009 and 2013, and bulk water samples from incubations in 2013, showed a consistent pattern of increasing ambient DIN and DIP from south to north that mirrors the larger surface chl *a* and temperature gradients across the SLI (Table 1). Malden and Starbuck were significantly enriched in DIN and DIP compared to Flint, Vostok and Millennium, regardless of whether the water was sampled from ~1 m above the benthos in 2009, throughout the water column in 2013, or from the nearshore surface water used in incubations (S2 Table). DIN was ~350% higher at Malden (4.63 ± 0.13 *μ*M) versus Flint (0.46 ± 0.01 *μ*M) in 2013, and ~200% higher than at either Vostok (0.93 ± 0.05 *μ*M) or Millennium (0.95 ± 0.08 *μ*M) (Table 1). Concentration of DIP was 75–150% higher at the northern islands than at the three southern islands (Table 1).

### Metabolism

**Fig 3:**
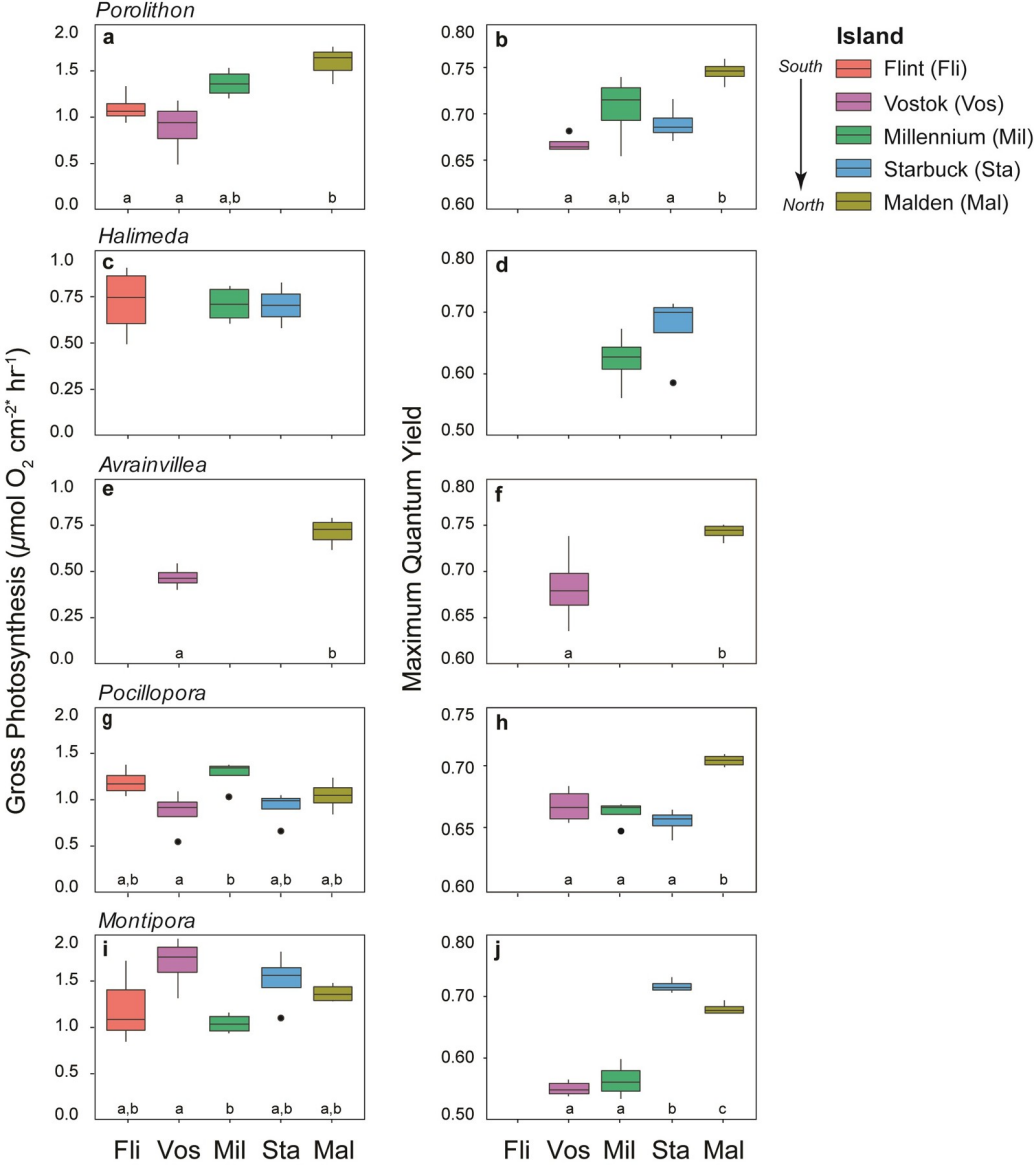
Coral and algal metabolism and photosynthetic efficiency. Box plots of gross photosynthesis and maximum quantum yield of (a-b) *Porolithon* sp., (c-d) *Halimeda* sp., (e-f) *Avrainvillea amadelpha*, (g-h) *Pocillopora meandrina*, and (i-j) *Montipora aequituberculata*. Islands are ordered from south to north across the x-axis (from left to right), and missing bars indicate where a given species was not present at an island. Due to logistical difficulties, there were no maximum quantum yield measurements at Flint or photosynthesis measurements from *Porolithon* at Starbuck. Significant differences between islands were determined by Tukey’s HSD, different letters represent significant differences at p < 0.05. Gross photosynthetic rates for *Porolithon* and corals were normalized to surface area, and are expressed as *μ*mol O_2_ cm^-2^ hr^-1^. *Rates for the algae, *Halimeda* and *Avrainvillea*, were normalized to fresh weight, thus for those taxa the units are *μ*mol O_2_ mg^-2^ hr^-1^.

Metabolic rates of all genera were variable across the SLI archipelago, and although there was some evidence of enhancement in ecophysiology with increasing proximity to the equator, the trend was not consistent across all taxa (Fig 3 and Table 2).

**Table 2:** Photosynthesis and respiration rates.

Genus	Island	Net Photosynthesis (*μ*mol O_2_ cm^-2^* hr^-1^)	Respiration (*μ*mol O_2_ cm^-2^* hr^-1^)
*Porolithon*	Flint	0.19 ± 0.16	0.91 ± 0.23
	Vostok	0.05 ± 0.13	0.94 ± 0.04
	Millennium	0.40 ± 0.15	0.96 ± 0.09
	Starbuck	-	0.44 ± 0.06
	Malden	0.59 ± 0.13	1.13 ± 0.05
Halimeda*	Flint	0.61 ± 0.07	0.11 ± 0.03
	Millennium	0.43 ± 0.05	0.28 ± 0.01
	Starbuck	0.42 ± 0.03	0.30 ± 0.04
Avrainvillea*	Vostok	0.15 ± 0.01	0.32 ± 0.03
	Malden	0.35 ± 0.05	0.36 ± 0.05
*Pocillopora*	Flint	0.69 ± 0.10	0.49 ± 0.03
	Vostok	0.34 ± 0.23	0.52 ± 0.06
	Millennium	0.66 ± 0.09	0.62 ± 0.04
	Starbuck	0.43 ± 0.13	0.49 ± 0.03
	Malden	0.49 ± 0.09	0.55 ± 0.02
*Montipora*	Flint	0.68 ± 0.17	0.62 ± 0.12
	Vostok	0.80 ± 0.09	0.90 ± 0.10
	Millennium	0.58 ± 0.05	0.45 ± 0.02
	Starbuck	0.93 ± 0.19	0.58 ± 0.02
	Malden	0.77 ± 0.08	0.60 ± 0.03

Mean (± SE) net photosynthesis and dark respiration rates of corals and algae surveyed across the SLI, *n* = 4 per genus at each island. Rates for *Porolithon* and corals were normalized to surface area.

*Rates for the algae, *Halimeda* and *Avrainvillea*, were normalized to fresh weight, thus, the units for those taxa are *μ*mol O_2_ mg^-2^ hr^-1^.

Photosynthesis (net and gross) and respiration of the CCA *Porolithon* were highest at Malden near the equator (Fig 3A and Table 2), but did not vary significantly across the three southern islands, Flint, Vostok, and Millennium (S3 Table). Some of the variability in gross photosynthesis is related to different patterns of response in net photosynthesis versus respiration, because gross photosynthesis is the sum of both. In *Porolithon*, the significant increase in gross photosynthesis at Malden is linked to a 211% increase in net photosynthesis from Flint to Malden, but only a 24% increase in respiration. In *Halimeda*, gross photosynthesis was consistent at the three islands where it was found (Flint, Millennium, Starbuck) (Fig 3C). However, this pattern obscures a marginally significant 45% increase in net photosynthesis at the southernmost island (Flint) (S3 Table), and a significant 173% increase in respiration rates at the northern islands (Table 2). These opposing trends in net photosynthesis and respiration across the islands are negated in the estimates of gross photosynthesis. In *Avrainvillea*, gross photosynthesis was 54% higher at Malden than Vostok (Fig 3E and Table 2), which was driven by significantly higher net photosynthesis (131%) but no difference in respiration from Vostok to Malden (S3 Table).

Coral metabolism was highly variable across the SLI. Gross photosynthesis of *Pocillopora* varied among islands but did not clearly relate to proximity to the equator and the associated increase in nutrient concentrations (Fig 3G and Table 2). These differences were associated with variable, but not significantly different, rates of net photosynthesis and respiration (S3 Table). The plating coral, *Montipora*, also had variable photosynthesis (net and gross) and respiration rates across the archipelago (Fig 3I and S3 Table), with highest rates at Vostok and lowest rates at Millennium (Table 2).

### Maximum quantum yield

Maximum quantum yield increased across the SLI with increasing proximity to the equator for all taxa, though the magnitude of change varied by genus. *Porolithon* maximum quantum yield was up to 65% higher at Malden than at the southern islands (Fig 3B), where yield did not differ significantly from Vostok and Millennium to Starbuck (S3 Table). Maximum quantum yield did not differ across islands for *Halimeda* (Fig 3D and S3 Table), and was significantly higher at Malden than at Vostok for *Avrainvillea* (Fig 3F and S3 Table).

Maximum quantum yield of the coral *Pocillopora* was significantly higher at Malden than at the southern islands, which did not differ from each other (Fig 3H and S3 Table). In *Montipora*, maximum quantum yield was significantly higher at Starbuck than at Malden, and higher at Malden than at Vostok and Millennium (Fig 3J and S3 Table).

### Photosynthetic pigments

**Fig 4:**
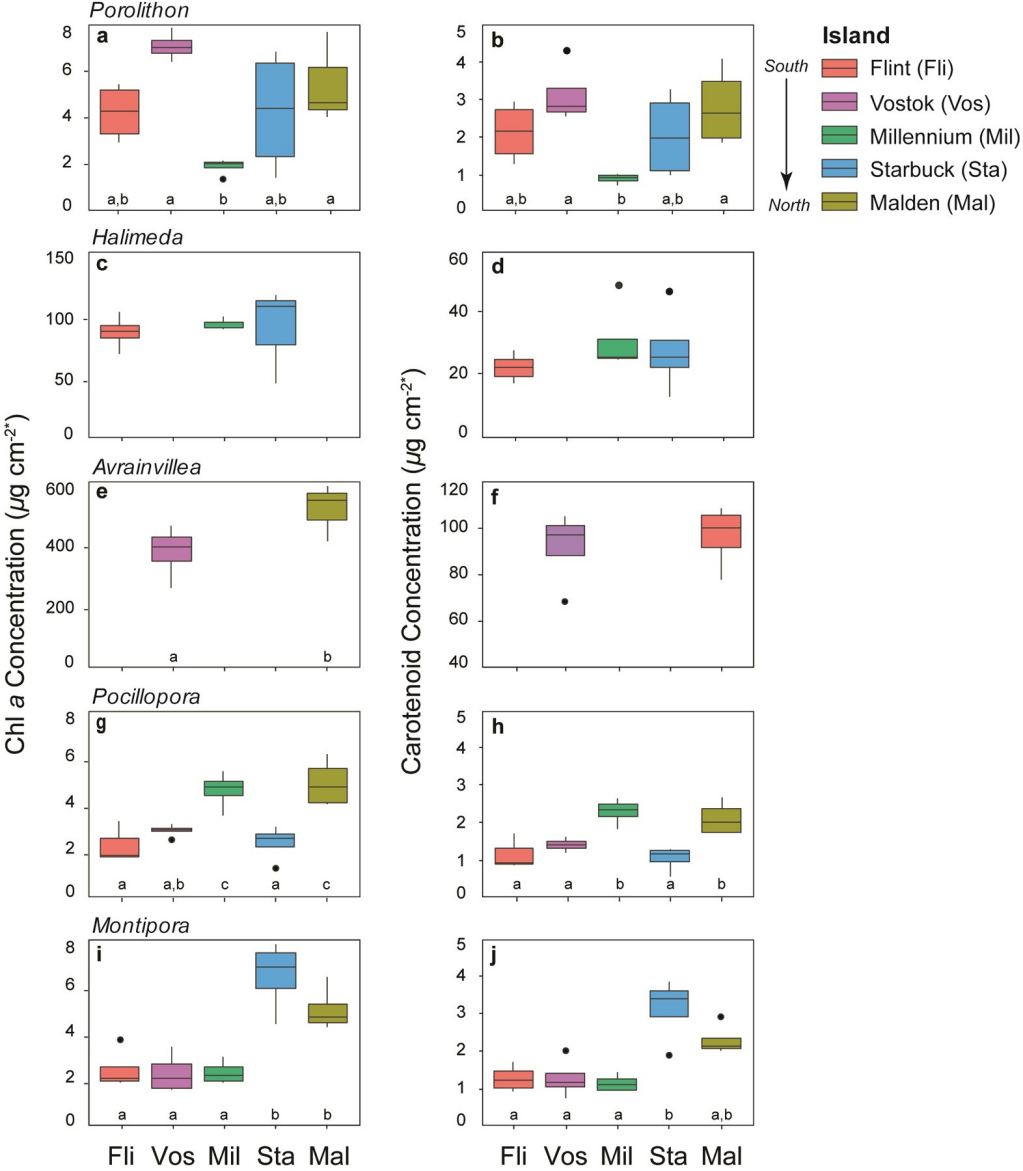
Photosynthetic pigments of corals and algae. Box plots of chl *a* and carotenoid concentrations in (a-b) *Porolithon* sp., (c-d) *Halimeda* sp., (e-f) *Avrainvillea amadelpha*, (g-h) *Pocillopora meandrina*, and (i-j) *Montipora aequituberculata*. Islands are ordered from south to north across the x-axis (from left to right), and missing bars indicate where a given species was not present at an island. Significant differences between islands were determined by Tukey’s HSD, different letters represent significant differences at p < 0.05. *Porolithon* and coral pigment concentrations were normalized to surface area, and rates are expressed as *μ*g cm^-2^*. *Halimeda and *Avrainvillea* pigment concentrations were normalized to fresh weight, thus units for those taxa are *μ*g mg^-2^.

Photosynthetic pigment concentrations generally followed the predicted pattern of increasing with proximity to the equator (Fig 4 and S3 Table). Chl *a* concentrations tended to increase from south to north for all taxa but *Porolithon* and *Halimeda*. However, in *Pocillopora*, chl *a* concentrations were lower than expected at Starbuck, but higher than expected at Millennium (Fig 4G). In *Montipora*, chl *a* concentrations increased by 115–198% from Flint to Malden (Fig 4I). Carotenoids showed little pattern across taxa or islands due to high levels of variation among individuals (Fig 4).

**Fig 5:**
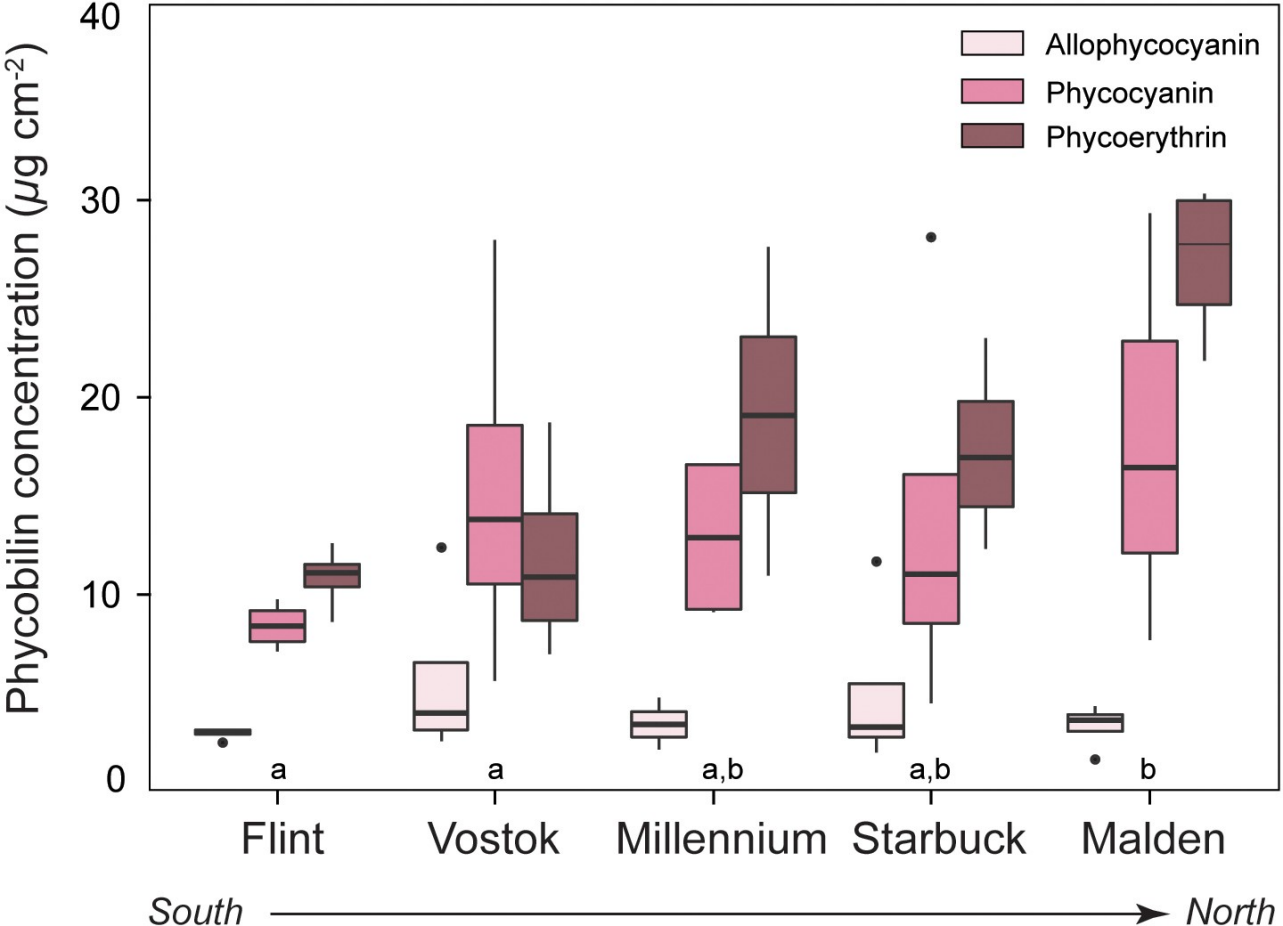
Phycobilin pigment concentrations. Mean (± SE) phycobilin pigment concentrations normalized to surface area for the crustose coralline alga *Porolithon* sp. across the Southern Line Islands. Islands are ordered from south to north. Allophycocyanin (light pink), phycocyanin (pink), and phycoerythrin (dark pink) were measured on 2 subsamples of each individual (*n* = 4) per island. Data points are means of subsamples per individual. Significant differences in pigment concentrations across islands were determined for each pigment with Tukey’s HSD. Different letters represent significant differences at p < 0.05.

Phycobilin pigment concentrations, including allophycocyanin, phycocyanin, and phycoerythrin of the coralline red alga *Porolithon* were variable across the SLI (Fig 5), with island-specific significant differences for phycoerythrin only (S3 Table). Phycoerythrin concentrations increased by 150% from Flint to Malden (Fig 5). Though there were similar trends for phycocyanin, including a 185% increase from Flint to Malden, these differences were not significant, likely due to high variability among individuals within an island.

## Discussion

The isolated and remote SLI represent a unique study system to evaluate how variability in exposure to equatorial upwelling, and thus availability of inorganic nutrients, influences coral and algal ecophysiology. Results of our in situ measurements, discrete water samples, and remote sensing of surface chl *a* illustrate that the SLI are increasingly exposed to high levels of surface primary production and inorganic nutrients with proximity to the equator. We hypothesized that the ecophysiology of benthic autotrophs (i.e., metabolism, maximum quantum yield, pigment concentrations) would reflect this pattern of nutrient enrichment. Although in some instances photophysiology was enhanced at the more nutrient-enriched islands, the overall patterns were variable across taxa and did not indicate a clear trend of physiological enhancement.

The lack of a clear relationship between exposure to upwelling and the ecophysiology of primary producers is notable because greater availability of inorganic nutrients generally has a predictable, positive effect on autotrophs. While our findings corroborate well-established patterns of increasing surface ocean primary production near the equator [10,56,57], they indicate that provisioning of nutrients from equatorial upwelling does not necessarily enhance autotroph physiology on the benthos, as we observed in the SLI. The ambiguous relationship between nutrient availability and ecophysiology suggests that other abiotic or biotic factors may be contributing to benthic biological processes in the central Pacific. These results indicate that nutrients may not be the primary drivers of benthic primary production on some coral reefs.

### Environmental parameters

The equatorial islands of Starbuck and Malden were cooler and more nutrient-rich than the southern islands of Flint, Vostok, and Millennium, which became incrementally warmer and depleted in nutrients with proximity to the equator. Our measurements of environmental conditions were constrained to discrete samples, short-duration in situ measurements, and short-term remote sensing of chl *a*. With these data, alone, our scope for interpretation is temporally limited. However, our findings corroborate a plethora of existing long-term data sets and a rich history of oceanographic research that, collectively, improve our understanding of long-term upwelling patterns in the remote equatorial Pacific. Decades of research have characterized equatorial upwelling as a persistent and predictable oceanographic feature driven by the Equatorial Undercurrent (EUC) [7,10]. The EUC delivers cool, nutrient rich water to the ocean surface on a west-to-east pathway [33], and causes net accumulation and temporal persistence of high nitrate and phosphate around the equator [34]. Higher DIN and DIP fuels phytoplankton growth and, thus, increases surface chl *a* concentrations. This trend is evident in our 4-month chl *a* data, and is even more conspicuous in the climatology from 2004–2015 [40]. Further, the same patterns have been described in numerous other studies in the same region [21,35,58]. The coupling of upwelling with oceanic production is well established, but much less is known about potential cascading effects on primary production of the benthos, particularly in coral reef habitats.

Coral reefs are typically considered to exist in oligotrophic waters that are often likened to marine deserts [24], and on average DIN and DIP concentrations in the Caribbean and Pacific are estimated to be ~0.4 *μ*M and ~0.2 *μ*M, respectively [59]. However, our findings demonstrate that nutrient concentrations on some reefs can well exceed these global averages. At the most equatorial site, Malden, the conditions are more akin to the nutrient regime of a temperate kelp forest in southern California, where nitrate concentrations can increase up to 5 *μ*M during wind-driven upwelling season [60], than the archetypal oligotrophic coral reef. Similar patterns of natural enrichment have been documented in other upwelling-influenced coral reef systems, such as on Conch Reef in the Florida Keys, where episodic pulses driven by internal tidal bores can increase ambient DIN to 1–4 *μ*M and DIP to 0.1–0.3 *μ*M [61]. Elevated levels of DIN and DIP often have negative implications for overall community structure on coral reefs, because excess nutrients can fuel the dominance of fleshy macroalgae [4]. Yet, the benthos on nutrient-enriched islands of Malden and Starbuck is dominated by reef-building corals and calcifying algae, where reef builders cover up to ~80% of the benthos (as opposed to fleshy and turf algae that account for ~15%) [37]. Our findings indicate that coral reef ecosystems in the central Pacific can thrive in nutrient conditions typically considered detrimental to reef-building corals. Indeed, coral cover is also high on Jarvis Island, which is just north of Malden and at the epicenter of equatorial upwelling, where DIN concentrations can naturally fluctuate up to 16 *μ*mol [62]. Though inorganic nutrients can influence benthic dynamics, the relative dominance of reef-builders on these islands likely is also linked to high biomass of herbivorous fishes [36]. Given the persistence of an extreme gradient in inorganic nutrient concentrations across coral reefs of the central Pacific, there is a clear need to explore the role of naturally nutrient variability in shaping biological processes and the resulting implications for the associated benthic reef communities.

### Metabolism

Evaluating the relationship between autotroph metabolism and inorganic nutrient availability provides insight into the potential effects of equatorial upwelling on benthic biological processes. Metabolic rates indicate the physiological status of corals and algae, and photosynthetic rates are directly proportional to rates of primary production. Given the potential for nutrient availability to influence rates of primary production in microalgae [63] and macrophytes [64], we hypothesized that photosynthesis of corals and algae would generally increase across the SLI corresponding to DIN and DIP availability. However, our measurements of coral and algal metabolism did not show a strong pattern of enhancement across the SLI, except in the CCA, *Porolithon*.

*Porolithon* metabolic rates generally tracked with the nutrient gradient. Of the benthic macroalgae, metabolism of the green alga, *Avrainvillea*, was higher at the site closer to the equator, while *Halimeda* was largely unchanged across sites. These data should be interpreted cautiously as each macroalgal genera was found at only 2 and 3 of the 5 islands, respectively. Metabolic rates of the corals, *Pocillopora* and *Montipora*, were variable across all islands with no particular pattern. The lack of a clear metabolic response to nutrient enrichment across all taxa in this study may indicate that other environmental factors influence metabolism, or may be a result of high variability associated with the methodology. Further, gross metabolic rates may not fully capture the capacity for autotrophs to respond to nutrient availability at the time-scale of the present study.

In contrast to photosynthetic efficiency (maximum quantum yield) and photosynthetic pigment content, which quantify the functioning of cellular light-harvesting machinery, metabolic rates constitute a net organismal response. Comparing and contrasting the metabolic responses of corals and macroalgae across the same gradient may thus be encumbered by inherent differences between strictly autotrophic algae and mixotrophic corals [65]. While algal metabolism represents cellular processes (e.g., respiration and photosynthesis) of only the autotrophic alga, coral metabolism is a net response of the coral holobiont, which encompasses the heterotrophic coral host, the autotrophic endosymbionts and the associated microbial community [66]. Estimating photosynthesis through oxygen production, particularly with corals, may be too coarse a metric to resolve finer-scale trends underlying genera-specific responses. The physiological effects of nutrient enrichment on corals may thus be more effectively assessed by pigment content and maximum quantum yield [67].

### Maximum quantum yield

Maximum quantum yield is a direct measure of the functioning of chl *a* pigment molecules, and provides a proxy for the photosynthetic efficiency of autotrophs. At the organismal scale, a higher photosynthetic efficiency indicates that, when all reaction centers are engaged, the autotroph can fix more carbon per quanta through the light-dependent reactions of photosynthesis [68]. Because photosynthetic efficiency is directly coupled with pigment content, and there is a predictable and well-established relationship between pigment content and nutrients, we hypothesized that maximum quantum yield would increase with increasing availability of DIN and DIP. Maximum quantum yield followed the predicted latitudinal pattern for corals and algae across the SLI, though the magnitude of response was taxon-specific.

Fluorescence measurements of photosystem II provide valuable insight into the photosynthetic performance of autotrophs and their response to varying nutrient regimes [69,70]. Indeed, photosynthetic efficiency of coral and algal taxa was highest at most northern islands sampled, and there was an overall trend of increasing photosynthetic efficiency with increasing exposure to equatorial upwelling. These results indicate that long-term exposure to ample DIN and DIP availability may increase the number, function, or efficiency of photosystem reaction centers. Our data corroborate a wide body of work demonstrating that DIN and DIP availability directly impacts the ability of algae to build and maintain properly functioning photosystem complexes that are essential for photosynthesis [20]. Enhanced photosynthetic efficiency could have ecological ramifications because efficiency is positively correlated with primary production [71,72]. Thus, greater exposure to inorganic nutrient availability may indicate higher potential for primary production and a potential increase in the capacity of benthic autotrophs to channel more energy to higher trophic levels. However, future experiments should confirm the consistency of these patterns by incorporating larger sample sizes and greater replication across natural nutrient regimes.

### Photosynthetic pigments

Pigment concentrations in algae and corals are flexible and highly responsive to prevailing environmental conditions [73], particularly to irradiance and nutrient concentrations [74]. A positive photosynthetic pigment-nutrient relationship has been demonstrated in a suite of photosynthetic taxa and across a range of environmental conditions. This relationship generally manifests as an increase in chl *a* and carotenoid concentrations in response to excess availability of DIN and DIP [14,75]. The predictability of this relationship makes algal pigment content a useful metric in understanding the abiotic conditions of an ecosystem because pigments in coral reef algae [76] and corals [67,77] can be used as bioindicators for nutrient pollution and water quality. Given the strength and consistency of this relationship, we expected that pigment concentrations would increase across the SLI with increasing proximity to the equator.

In most taxa studied, the primary photosynthetic pigments, chl *a* and carotenoids, were highest at the most northern islands, with some variability across the middle islands of the archipelago. Chl *a* generally increased with greater exposure to nutrient availability in the green algae and the corals, and patterns of carotenoids mirrored those of chl *a*, but with more variability. *Porolithon* showed the least consistent pattern with respect to the primary pigments, but the accessory phycobilin pigments demonstrated the strongest response to the nutrient gradient. There was a positive, incremental increase in phycoerythrin and phycocyanin concentrations at islands that corresponded to increasing availability of ambient DIN and DIP, though this trend was significant only for phycoerythrin. The phycobilin complexes in crustose corallines, and other red algae, act as accessory pigments that broaden the spectrum of light available for photosynthesis [78]. Phycobilins are also particularly responsive to DIN, in part because algae can use phycobilin pigment complexes to store excess nitrogen [54]. Nitrogen storage for luxury consumption by algae is an ecological adaptation to nutrient limitation and provides a source of DIN that can be later metabolized to fuel growth when nutrients are limiting [79]. The increase in *Porolithon* phycobilins with greater exposure to inorganic nutrient availability may indicate that these algae can store the excess DIN delivered by equatorial upwelling in pigment-protein complexes. The increase in some of the photosynthetic pigments with increased availability of DIN and DIP indicates that equatorial upwelling could fuel primary production in benthic autotrophs by facilitating the development of more pigments, which supports greater capacity to harvest light and generate energy.

### Effects of equatorial upwelling on ecophysiology

The lack of a strong relationship between benthic autotroph ecophysiology and nutrient availability in the SLI is an unexpected result, and indicates that DIN and DIP may not have been the only limiting factor for metabolism and photophysiology in the taxa studied. Other potential abiotic drivers of autotroph ecophysiology across islands could include temperature, light, and other nutrients. For many algae, maximum photosynthesis occurs over a range of temperatures, thus the 1.5°C change in temperature from south to north did not likely contribute to metabolic rates [80]. Similarly, light can be ruled out as a confounding factor because there was no significant difference in the amount of available light across the SLI, despite the 4° change in latitude. Biotic factors could also have influenced ecophysiology as well, such as prior damage from grazing or exposure to competition or disease, though we attempted to minimize these effects by selecting individuals that appeared healthy.

The limited temporal scope of both our physiological and environmental measurements are also important to consider. Due to the logistical constraints associated with field work at remote islands, physiological measurements could only be taken once, and thus represent a snapshot of ecophysiology at one point in time. The inorganic nutrient gradient resulting from upwelling is persistent through time [40], but could be magnified at different times of the year, depending on the strength of prevailing winds. Our environmental measurements are likewise temporally limited, though we have bolstered our discrete samples with historical perspective and satellite data that encompasses several months around the expedition. Furthermore, we quantified only DIN and DIP in our water samples, using discrete samples at one time point over two different expeditions. Thus, we cannot conclude what other nutrients may have influenced autotroph ecophysiology. We can speculate that iron limitation of photosynthesis may have contributed to some of the observed patterns in photosynthesis (and photophysiology). In areas of high nitrate and low surface chl *a*, iron availability can limit photosynthesis and growth of phytoplankton [81]. However, our satellite data illustrate that there was high surface-ocean production at the equatorial islands, and, thus, do not provide evidence that phytoplankton primary production was iron limited. Future work should explore the relationship of coral and algal ecophysiology in response to equatorial upwelling in more detail, the potential for seasonal responses to inorganic nutrient availability, and the role of additional abiotic factors in constraining benthic photosynthesis in nutrient replete conditions.

These data provide an interesting perspective on the role of inorganic nutrients and upwelling in shaping benthic biological processes. Despite the potential for nutrient availability to elicit strong photosynthetic responses, we found only moderate, and sometimes inconsistent, physiological enhancement with increasing exposure to equatorial upwelling in the taxa studied. This lack of a clear pattern contrasts with the conspicuous gradient in surface ocean productivity and inorganic nutrient availability. Our findings agree with one other study to explore ecophysiology of coral reef benthic taxa in response to upwelling. Eidens et al. (2014) similarly documented substantial variation in benthic autotroph metabolic responses across an upwelling gradient in Colombia [82]. Using comparable methodologies, they found that upwelling increased photosynthetic rates in turf algae, decreased rates in corals and inconsistently affected photosynthesis in macroalgae and coralline algae [82]. Though surface ocean productivity is key in the biophysical coupling of large-scale oceanography with benthic communities, it does not appear to be directly linked to patterns in benthic autotroph ecophysiology, at least in short-term physiological assessments. Future experiments should incorporate growth rates, net community metabolism, and other more integrated measures of benthic production to better understand the relationship between surface ocean production and benthic ecosystem processes.

## Conclusions

Here we document, for the first time, the ecophysiology of abundant benthic autotrophs on coral reefs of the SLI in the remote central Pacific. We illustrate that the SLI span a conspicuous gradient of increasing surface ocean production and inorganic nutrient availability (DIN, DIP) that indicate the SLI become increasingly exposed to upwelling with decreasing distance to the equator (i.e., latitude). Though we predicted that coral and algal metabolism (photosynthesis, respiration) and photophysiology (maximum quantum yield, photosynthetic pigments) would increase with greater exposure to upwelling, we found evidence of this in only some parameters. The CCA, *Porolithon*, generally responded positively with greater exposure to nutrient availability, particularly with respect to metabolism and phycobilin pigment content. The green algae and corals also showed some indications of photophysiological enhancement, but with more variability. The lack of a strong and consistent physiological response in all taxa across this upwelling gradient indicates that other factors may be limiting photosynthesis and photophysiology of benthic autotrophs in the SLI.

The effects of nutrients on organismal physiology may have broader ecosystem implications, if physiological enhancement (or lack thereof) is indicative of primary production. Our results would then suggest that benthic primary production may not always track with patterns in surface ocean productivity. A limited number of studies on upwelling in coral reefs indicate that inorganic nutrient delivery via upwelling or internal tides can increase surface ocean productivity of oceanic islands and atolls [32], biomass of fish [2], and heterotrophy in corals [40]. However, the link between increased surface productivity due to upwelling and benthic productivity remains unclear. Unraveling the role of natural nutrient sources in shaping coral reefs may be one key to improving our understanding of how coral reef community structure and function will change as humans continue to alter natural nutrient landscapes.

## Supporting information

S1 Table Coordinates, land area, and benthic taxa sampled at the five Southern Line Islands in the Republic of Kiribati. Islands closest to the equator have higher inorganic nutrient concentrations due to equatorial upwelling. In all tables and figures, islands are listed from south to north in order of increasing proximity to the equator.
(DOCX)

S2 Table ANOVA table of environmental conditions by island. Significance at p < 0.05 is noted in bold.
(DOCX)

S3 Table ANOVA table of island effects on ecophysiology by genera. Significance at p < 0.05 is noted in bold.
(DOCX)

S4 Table Raw physiological data of corals and algae across the Southern Line Islands. Raw physiological data of coral and algae replicates from incubations, quantum yield measurements, and pigment content.
(XLSX)
