# Supplementary material for: Ecophysiology of coral reef primary producers across an upwelling gradient in the tropical central Pacific
Source: PLoS One. 2020 Feb 4;15(2):e0228448. doi: 10.1371/journal.pone.0228448 (PMC6999896; doi:10.1371/journal.pone.0228448)
Supplement: S1 Table — Islands closest to the equator have higher inorganic nutrient concentrations due to equatorial upwelling. In all tables and figures, islands are listed from south to north in order of increasing proximity to the equator. (DOCX) [file pone.0228448.s001.docx]

**Supporting Information**

**S1 Table. Coordinates, land area, and benthic taxa sampled at the five Southern Line Islands in the Republic of Kiribati.**

Islands closest to the equator have higher inorganic nutrient concentrations due to equatorial upwelling. In all tables and figures, islands are listed from south to north in order of increasing proximity to the equator.

| Island | Land (km^2^) | Coordinates | Coral | Algae |
| --- | --- | --- | --- | --- |
| Flint | 3.2 | 11°26’S 151°48’W | *Pocillopora*  *Montipora* | *Porolithon Halimeda* |
| Vostok | 0.24 | 10°06’S 152°25’W | *Pocillopora*  *Montipora* | *Porolithon Avrainvillea* |
| Millennium | 3.76 | 9°57’S  150°13’W | *Pocillopora*  *Montipora* | *Porolithon Halimeda* |
| Starbuck | 16.2 | 5°37’S  155°56’W | *Pocillopora*  *Montipora* | *Porolithon Halimeda* |
| Malden | 39.3 | 4°01’S  154°59’W | *Pocillopora*  *Montipora* | *Porolithon Avrainvillea* |
