# Supplementary material for: Ecophysiology of coral reef primary producers across an upwelling gradient in the tropical central Pacific
Source: PLoS One. 2020 Feb 4;15(2):e0228448. doi: 10.1371/journal.pone.0228448 (PMC6999896; doi:10.1371/journal.pone.0228448)
Supplement: S2 Table — Significance at p < 0.05 is noted in bold. (DOCX) [file pone.0228448.s002.docx]

**Supporting Information**

**S2 Table. ANOVA table of environmental conditions by island.**

Significance at p < 0.05 is noted in bold.

| Parameter | *F* | *p* |
| --- | --- | --- |
| Satellite chl *a* | 29.41 | **< 0.001** |
| Temperature | 266.70 | **< 0.001** |
| Light | 1.307 | 0.354 |
| DIN  2013 incubation | 119.83 | **< 0.001** |
| 2013 in situ | 568.08 | **< 0.001** |
| 2009 | 24.47 | **< 0.001** |
|  |  |  |
| DIP  2013 incubation | 15.35 | **< 0.001** |
| 2013 in situ | 136.28 | **< 0.001** |
| 2009 | 10.85 | **< 0.001** |
