# Supplementary material for: Ecophysiology of coral reef primary producers across an upwelling gradient in the tropical central Pacific
Source: PLoS One. 2020 Feb 4;15(2):e0228448. doi: 10.1371/journal.pone.0228448 (PMC6999896; doi:10.1371/journal.pone.0228448)
Supplement: S3 Table — Significance at p < 0.05 is noted in bold. (DOCX) [file pone.0228448.s003.docx]

**Supporting Information**

**S3 Table. ANOVA table of island effects on ecophysiology by genera.**

Significance at p < 0.05 is noted in bold.

| Response | *Porolithon* | | *Halimeda* | | *Avrainvillea* | | *Pocillopora* | | *Montipora* | |
| --- | --- | --- | --- | --- | --- | --- | --- | --- | --- | --- |
|  | *F* | *p* | *F* | *p* | *F* | *p* | *F* | *p* | *F* | *p* |
| Gross photosynthesis | 8.59 | **0.002** | 0.02 | 0.983 | 25.86 | **0.002** | 3.66 | **0.029** | 3.64 | **0.031** |
| Net photosynthesis | 3.85 | **0.039** | 4.40 | 0.052 | 35.50 | **0.001** | 2.33 | 0.103 | 1.81 | 0.182 |
| Dark respiration | 4.57 | **0.013** | 13.38 | **0.002** | 0.73 | 0.425 | 1.86 | 0.170 | 5.65 | **0.006** |
| Maximum  quantum yield | 8.66 | **0.002** | 1.89 | 0.219 | 7.64 | **0.033** | 17.98 | **< 0.001** | 99.37 | **< 0.001** |
| Chl *a* | 4.97 | **0.009** | 0.61 | 0.566 | 6.94 | **0.039** | 9.85 | **< 0.001** | 15.20 | **< 0.001** |
| Carotenoids | 3.91 | **0.023** | 0.67 | 0.534 | 0.20 | 0.669 | 9.38 | **< 0.001** | 10.74 | **< 0.001** |
| Phycocyanin | 1.47 | 0.260 | - | - | - | - | - | - | **-** | **-** |
| Phycoerythrin | 7.22 | **0.002** | - | - | - | - | - | - | - | - |
| Allophycocyanin | 0.68 | 0.615 | - | - | - | - | - | - | - | - |
